# Surface Modifications of Dental Ceramic Implants with Different Glass Solder Matrices: *In Vitro* Analyses with Human Primary Osteoblasts and Epithelial Cells

**DOI:** 10.1155/2014/742180

**Published:** 2014-09-14

**Authors:** Jana Markhoff, Enrico Mick, Aurica Mitrovic, Juliane Pasold, Katharina Wegner, Rainer Bader

**Affiliations:** ^1^Department of Orthopaedics, Research Lab for Biomechanics and Implant Technology, University Medicine Rostock, Doberaner Straße 142, 18057 Rostock, Germany; ^2^ZM Praezisionsdentaltechnik GmbH, Breite Straße 16, 18055 Rostock, Germany

## Abstract

Ceramic materials show excellent esthetic behavior, along with an absence of hypersensitivity, making them a possible alternative implant material in dental surgery. However, their surface properties enable only limited osseointegration compared to titanium implants. Within this study, a novel surface coating technique for enhanced osseointegration was investigated biologically and mechanically. Specimens of tetragonal zirconia polycrystal (TZP) and aluminum toughened zirconia (ATZ) were modified with glass solder matrices in two configurations which mainly consisted of SiO_2_, Al_2_O_3_, K_2_O, and Na_2_O. The influence on human osteoblastic and epithelial cell viability was examined by means of a WST-1 assay as well as live/dead staining. A C1CP-ELISA was carried out to verify procollagen type I production. Uncoated/sandblasted ceramic specimens and sandblasted titanium surfaces were investigated as a reference. Furthermore, mechanical investigations of bilaterally coated pellets were conducted with respect to surface roughness and adhesive strength of the different coatings. These tests could demonstrate a mechanically stable implant coating with glass solder matrices. The coated ceramic specimens show enhanced osteoblastic and partly epithelial viability and matrix production compared to the titanium control. Hence, the new glass solder matrix coating could improve bone cell growth as a prerequisite for enhanced osseointegration of ceramic implants.

## 1. Introduction

Titanium and titanium alloys are widely used materials in dental and orthopedic replacement surgery. However, mechanical benefits, excellent biocompatibility, early osseointegration, and high corrosion resistance due to the titanium passivation oxide layer [1–4] are accompanied by the dark grey color, the gingiva eventually becoming translucent, and tissue discoloration, as well as allergic reactions and sensitivities [5–7]. Over the years, ceramics were also proven to be an adequate alternative implant material. In particular, oxide ceramics such as alumina (Al_2_O_3_) and zirconia (ZrO_2_) enable promising osseointegration with concurrent mechanical and chemical stability, and wear and corrosion resistance are representative [8–10]. Furthermore, reduced bacterial adhesion and less plaque enrichment [9, 11], as well as a low allergic potential and esthetic appearance, indicate superiority in the field of dentistry [5, 7]. Cell adherence and thereby growth, migration, and differentiation are crucial and are influenced by implant material and surface topography [4, 12–14]. To date,* in vitro* experiments have shown controversial results with respect to increased cell behavior on either smooth or roughened titanium or ceramic implant surfaces [7, 15–17]. For improved osseointegration and secondary stability, bioglass coatings [18–20] and various implant surface modification techniques like sandblasting, acid etching, and titanium plasma spray were used [1, 4, 21].

In a preliminary study, the surface modification of ceramics with glass solder matrices was investigated with respect to the mechanical properties surface roughness and adhesive strength [22]. The present study aimed to investigate the cell biological response of human osteoblasts and gingival epithelial cells to ceramic implants coated with two different glass solder matrices. Furthermore, surface roughness, adhesive strength, and bending strength of the different glass solder matrices should be evaluated.

## 2. Materials and Methods

### 2.1. Specimens

Ceramic specimens manufactured by Metoxit AG (Thayngen, Switzerland), according to DIN EN 60267, were used for the present investigations. On the one hand, tetragonal zirconia polycrystal with alumina (TZP-A), consisting of ZrO_2_, Y_2_O_3_, and Al_2_O_3_ at levels of approximately 95%, 5%, and 0.25%, was utilized, while, on the other hand, alumina-toughened zirconia (ATZ) with approximately 76% ZrO_2_, 20% Al_2_O_3_, and 4% Y_2_O_3_ was tested. Two different kinds of samples were fabricated: Ø10 × 5 mm pellets were fabricated for roughness and adhesive strength testing as well as for all cell biological investigations; bending tests were performed on ceramic rods with dimensions of Ø4.3 × 60 mm.

### 2.2. Surface Modification

The surfaces of the ceramic specimens were modified with glasses of silica-based materials taken from the DCM hotbond series [23], which can be applied for the conditioning of mixed ceramics or pure zirconia. They mainly contain SiO_2_ (60–70%), Al_2_O_3_ (4–10%), K_2_O (6–10%), and Na_2_O (6–10%). Two different configurations of the glass matrix were applied to the ceramics: S1 and S2 (equal to HT1 in [22]) with grit sizes of 9.2 *μ*m and 12.6 *μ*m, respectively, and curing temperatures of 1000°C and 1035°C, respectively. The application procedure is described in detail in [22]. Subsequent to that, the glass surfaces were sandblasted with corundum (Al_2_O_3_) at a jet pressure of 1 bar and cleaned via an ultrasonic bath (distilled water) afterwards. The pellets were coated on their cylindrical end faces and the rods were modified circumferentially.

### 2.3. Mechanical Investigations

#### 2.3.1. Surface Roughness

The surface topography of bilaterally coated TZP-A pellets was determined using a profilometer (Hommel-Etamic T1000, Jenoptik AG, Jena, Germany). Surface roughness parameters (*R*_*a*_ and *R*_*z*_) were recorded performing threefold (0°, 60°, and 120°) line scans on each coated surface. The corresponding results were referenced to untreated TZP-A, sandblasted TZP-A, and sandblasted titanium.

Furthermore, field emission scanning electron microscopy (FESEM) was conducted using the MERLIN VP Compact microscope (Carl Zeiss, Jena, Germany) to evaluate modification of the surface structure of the TZP-A pellets by two different configurations of glass matrix in comparison to untreated TZP-A and sandblasted titanium specimens.

#### 2.3.2. Adhesive Strength

Determination of the adhesive strength of the glass solder matrices was performed consistent with Mick et al. [22]. Bilaterally coated TZP-A pellets were bonded to sandblasted titanium (grade V) cylinders using HTK Ultra Bond 100 (HTK Hamburg GmbH, Hamburg, Germany). A universal testing machine (Z050, Zwick GmbH & Co. KG, Ulm, Germany) was used for performing pull-off tests at a crosshead speed of 5 mm/min. The maximum force was measured and converted into the adhesive strength of the particular surface modification.

#### 2.3.3. Bending Strength

**Figure 1:**
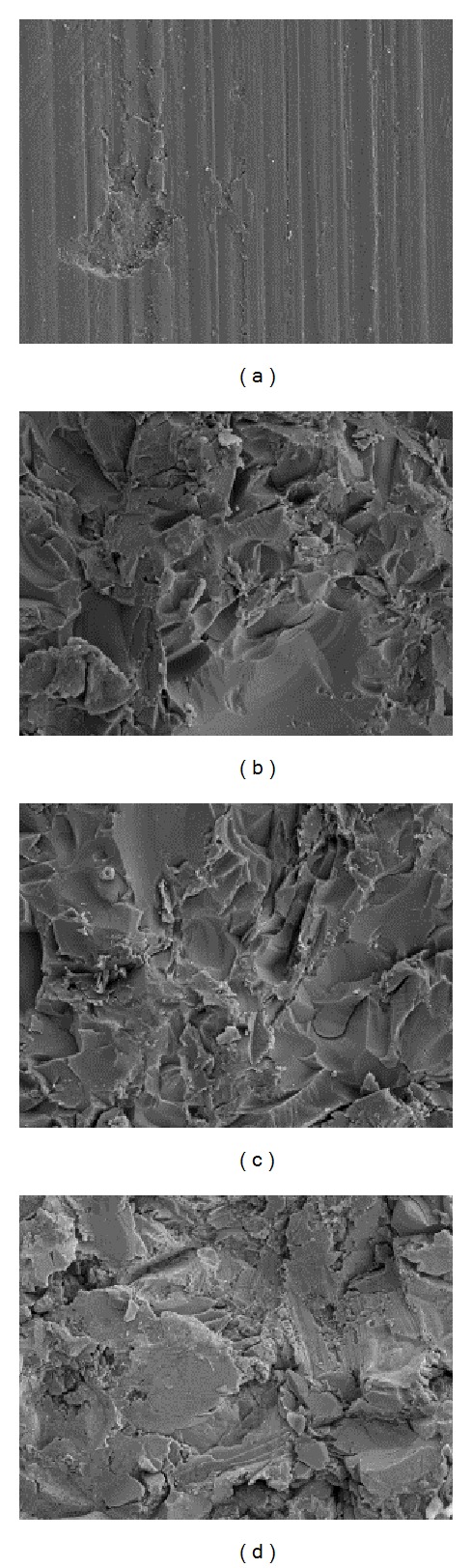
Field emission scanning electron microscopy images of different specimen surfaces. (a) Native TZP-A; (b) TZP-A with glass matrix S1; (c) TZP-A with glass matrix S2; (d) rough titanium. Magnification = 1000x.

In order to determine the influence of the coating procedure on the bending strength of the ceramic base bodies, 4-point-bending tests were performed on ceramic rods in the style of EN 843-1. The abovementioned universal testing machine Z050 was equipped with custom-made bearings with a support span of 40 mm and a loading span of 20 mm (see Figure 1), ensuring a constant bending moment between the load bearings. The crosshead speed was set to 0.7 mm/min, enabling failure of the rod within 60 seconds. The bending strength of each sample was derived from the maximum force and geometry of the test setup. TZP-A and ATZ rods were tested in native, sandblasted, and coated conditions. Furthermore, some samples were also tested after burning without any glass matrix on the surface to investigate the influence of mere tempering on the bending strength of TZP-A.

### 2.4. Cell Biological Investigations

#### 2.4.1. Isolation and Cultivation

Isolation of human primary osteoblasts followed a previously described procedure [24]. The samples for the* in vitro* experiments were collected after patient agreement had been obtained. The study was approved by the Local Ethical Committee (registration number: A2010-10). Human primary osteoblasts were isolated from the spongiosa of the femoral heads of patients undergoing primary total hip replacement. Cells were cultivated in osteogenic cell culture medium (Modified Eagle's Medium Dulbecco (Biochrom AG, Berlin, Germany)) containing 10% FCS, 1% penicillin/streptomycin, 1% amphotericin B, and 1% HEPES buffer (all: Gibco-Invitrogen, Darmstadt, Germany), as well as the osteogenic additives dexamethasone (100 mM), L-ascorbic acid (50 *μ*g/mL), and *β*-glycerophosphate (10 mM) (all: Sigma-Aldrich, Munich, Germany) until they reached confluence. The osteogenic character of the isolated cells was verified by conducting alkaline phosphatase staining using a fuchsin + substrate chromogen (DAKO, Hamburg, Germany).

The gingival epithelial cell line Ca9-22 was ordered from the Leibniz Institute DSMZ—German Collection of Microorganisms and Cell Cultures (Brunswick, Germany). Cell cultivation was performed using Dulbecco's Modified Eagle's Medium (DMEM) supplemented with 10% FCS until cells reached confluence.

Human osteoblasts in the second passage and gingival epithelial cells (20,000 cells/500 *μ*L each) were transferred to native ceramic specimens (TZP-A, ATZ) and ceramic specimens coated with glass solder matrices S1 and S2, as well as titanium specimens (*R*_*z*_ = 20 *μ*m) as a control.

#### 2.4.2. Analyses of Cell Viability and Matrix Production

After 96 hours of cultivation, metabolic activity of osteoblasts and epithelial cells, respectively, was determined via mitochondrial dehydrogenase activity (WST-1) (Roche, Grenzach-Wyhlen, Germany). Thereby, the tetrazolium salt WST is transformed to formazan by mitochondrial succinate dehydrogenase from the metabolically active cells. The adsorption was measured at 450 nm in a microplate reader (Opsys MRTM, Dynex Technologies GmbH, Denkendorf, Germany) and was found to be directly proportional to the metabolic cell activity. Qualitative cell viability was analyzed by means of live/dead staining with the two fluorescence dyes calcein AM for vital cells and ethidium homodimer-1 for dead ones (Live/Dead cell viability assay, Invitrogen, Darmstadt, Germany). An enzyme-linked immunosorbent assay (Metra C1CP EIA Kit, Quidel, Buende, Germany) was used to verify the synthesis of procollagen type 1 in the osteoblasts.

#### 2.4.3. Statistical Analysis

The statistical significance of all data was evaluated by ANOVA post hoc LSD using IBM SPSS Statistics Version 20 (IBM Corp., New York, USA). Significance level was set to *P* < 0.05.

## 3. Results

### 3.1. Mechanical Investigations

The average surface roughness *R*_*z*_ of TZP-A samples modified with glass solder matrix S1 (20.56 ± 2.31 *μ*m) was significantly higher than that of the references native TZP-A (1.57 ± 0.16 *μ*m; *P* < 0.001) and sandblasted TZP-A (4.28 ± 0.61 *μ*m; *P* < 0.001) but lower than sandblasted titanium (22.82 ± 0.61 *μ*m; *P* = 0.006). S2 showed significantly higher average surface roughness (20.44 ± 1.23 *μ*m [22]) compared to native and sandblasted TZP-A (each *P* < 0.001), which was lower than sandblasted titanium (*P* = 0.008). The glass solder matrices S1 and S2 did not differ significantly in average surface roughness (*P* > 0.05). The mean roughness index, *R*_*a*_, showed the same tendencies and differences when comparing S1 (3.49 ± 0.20 *μ*m) and S2 (3.61 ± 0.23 *μ*m) to each other (*P* > 0.05) and to the reference surfaces of native TZP-A (0.20 ± 0.03 *μ*m) and sandblasted TZP-A (0.65 ± 0.08 *μ*m) (*P* < 0.001). However, there were no significant differences in mean roughness index when comparing S1 and S2 to sandblasted titanium (3.63 ± 0.08 *μ*m; *P* > 0.05). Exemplary field emission scanning electron microscopy images of native and modified TZP-A specimens as well as titanium surfaces are shown in Figure 1.

**Figure 2:**
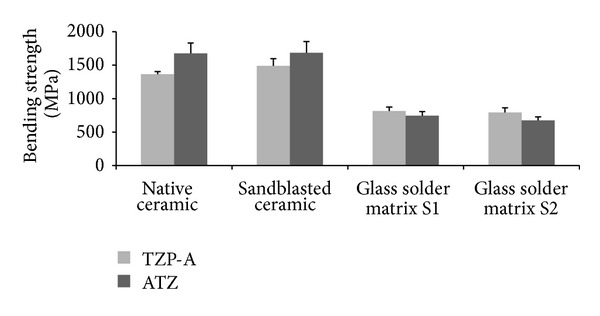
Bending strength of native ceramics, sandblasted ceramics, and ceramics after surface modifications (S1 and S2); *n* = 5 for TZP-A and *n* = 10 for ATZ.

Adhesive strength of the surface modification S1 was determined as 73.2 ± 7.2 MPa showing no significant difference in comparison to S2 (72.4 ± 11.8 MPa [22]; *P* > 0.05). Bending strength of native TZP-A rods (1365 ± 40 MPa) was raised significantly to 1491 ± 106 MPa due to sandblasting (*P* = 0.027). Coating with S1 led to a significant decrease (815 ± 58 MPa; *P* < 0.001). S2 showed significantly lower bending strength (793 ± 71 MPa) compared to native TZP-A (*P* < 0.001). Sandblasted ATZ specimens (1684 ± 169 MPa) did not have a significantly different bending strength than those under native conditions (1674 ± 157 MPa; *P* > 0.05). Surface modification with S1 (743 ± 62 MPa) and S2 (674.6 ± 53 MPa) resulted in significantly lower bending strength compared to native ATZ (each *P* < 0.001). For both ceramics, S1 and S2 did not differ significantly in bending strength (each *P* > 0.05).  Figure 2 shows the corresponding results.

Furthermore, the comparison of native and heated sandblasted TZP-A revealed a significant decrease in bending strength to 1075 ± 82 MPa after heating (*P* < 0.001).

### 3.2. Cell Activity and Viability

**Figure 3:**
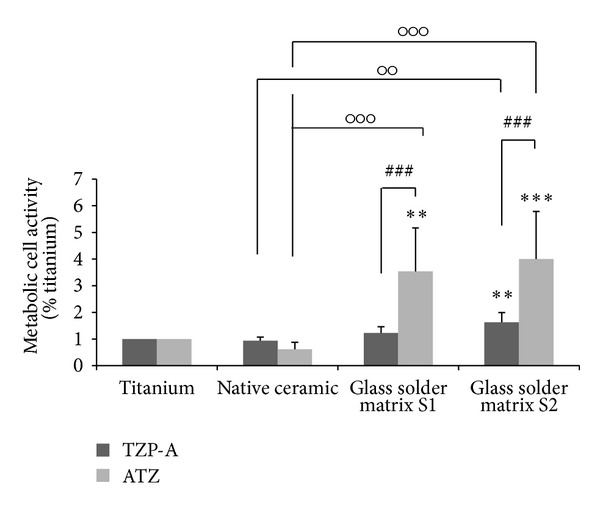
Metabolic activity of human osteoblasts cultured for 96 hours on specimens with different surface properties. Values are means ± SD (TZP-A *n* = 15; ATZ *n* = 8). Statistical significance levels (**P ≤ 0.01; ***P ≤ 0.001) compared to titanium (∗), between the native and modified ceramics (∘) and the relative ceramic types (#).

Influence of oxide ceramics (TZP-A, ATZ) with two different glass solder matrix coatings on human osteoblasts and gingival epithelial cell lines was determined. Analysis of metabolic activity after 96 hours revealed a nonsignificant increased activity of human osteoblasts cultured on both TZP-A and ATZ ceramics with the S1 glass solder matrix compared to the titanium control and the native ceramic (Figure 3). Moreover, the glass solder matrix S2 with a higher grit size (12.6 *μ*m) and burned with a higher curing temperature (1035°C) resulted in a significant increase of metabolic cell activity in contrast to the titanium control (TZP-A: *P* = 0.006; ATZ: *P* ≤ 0.001) and the native ceramics (TZP-A: *P* = 0.002; ATZ: *P* ≤ 0.001). Metabolic activity was significantly increased on ATZ with the glass solder matrix S1 compared to titanium (*P* = 0.002) and the native ceramic (*P* = 0.001). Furthermore, cell activity was significantly higher on ATZ over TZP-A (both: *P* ≤ 0.001). At least, data indicate a slightly decreased cell viability of osteoblasts cultured on the native ceramic specimens compared to titanium.

**Figure 4:**
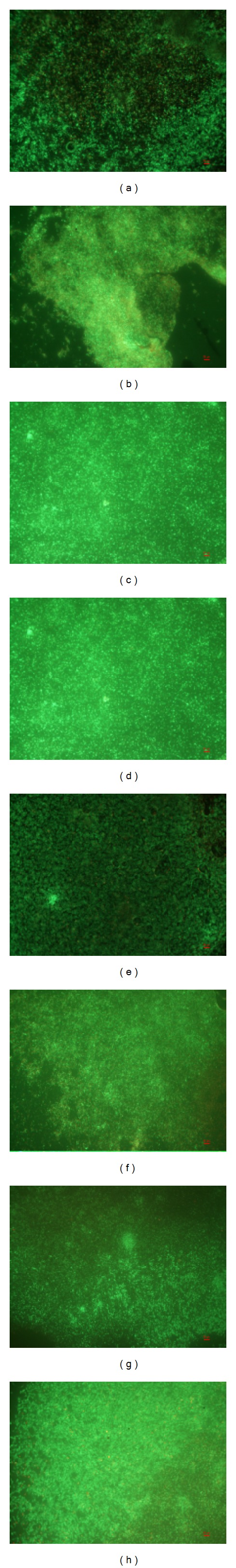
Live/dead staining of human osteoblasts cultured for 96 hours on specimens with different surface properties. (a)/(e) Titanium; (b) native TZP-A ceramic; (c) TZP-A with glass solder matrix S1; (d) TZP-A with S2; (f) native ATZ ceramic; (g) ATZ with S1; (h) ATZ with S2. Living cells are displayed in green and dead ones in red. Scale bar: 50 *μ*m.

Live/dead staining was conducted after 96 hours of cultivation to obtain a qualitative overview of bone cell viability. Thereby, living bone cells on each surface were displayed in large areas (Figure 4). Cells on the native ceramics also exhibit unsettled areas. Osteoblasts on the glass solder matrix S2 showed the highest metabolic activity in the WST-1 test.

**Figure 5:**
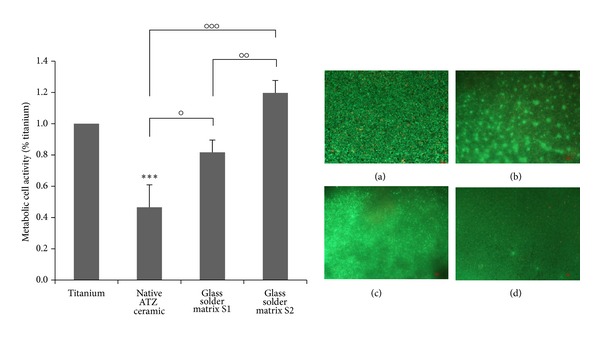
Left: metabolic activity of the gingival epithelial cell line Ca9-22 cultured for 96 hours on specimens with different surface properties. Values are means ± SD (*n* = 4). Statistical significance levels (°*P* ≤ 0.05; ^∘∘^ *P* ≤ 0.01; ***P < 0.001) compared to titanium (∗) and between the native and the modified ceramics (∘). Right: Live/dead staining of Ca9-22 cells cultured on (a) native titanium, (b) native ATZ ceramic, (c) ATZ with glass solder matrix S1, and (d) ATZ with glass solder matrix S2. Living cells are displayed in green and dead ones in red. Scale bar: 50 *μ*m.

Additionally, the gingival compatibility of the coated ceramic materials was proven with a gingival epithelial cell line (Ca9-22). Thereby, cell viability was decreased on the native and S1 coated ceramic specimens compared to titanium. Cell viability on ATZ with the glass solder matrix S2 was significantly increased compared to all other ceramic specimens (Figure 5). Live/dead staining showed large areas of cells on all specimens which were especially dense on titanium and ATZ with the glass solder matrix S2.

### 3.3. Collagen Synthesis

**Figure 6:**
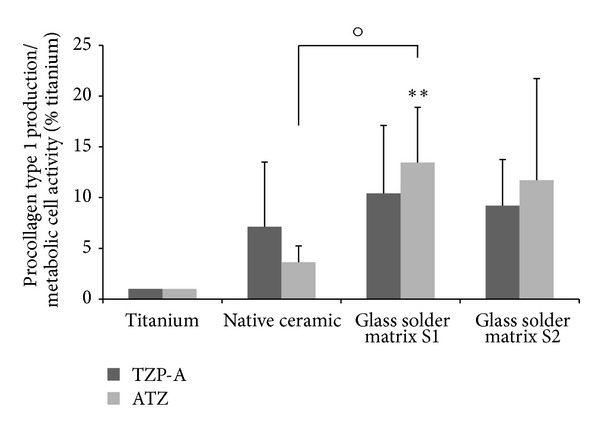
Synthesis of procollagen type 1 of human osteoblasts cultured for 96 hours on specimens with different surface properties. Values are means ± SD (TZP-A *n* ≥ 4; ATZ *n* ≥ 5). Statistical significance levels (°*P* ≤ 0.05; **P ≤ 0.01) compared to titanium (∗) and between the native and modified ceramics (∘).

The measurement of procollagen type 1 synthesis revealed an increased matrix production for all ceramic surfaces compared to titanium (Figure 6). Thereby, synthesis on the glass solder matrix S1 on ATZ was significantly higher than on titanium (*P* = 0.008) and the native ceramic specimens (*P* = 0.049).

## 4. Discussion

The application of ceramic implants in the field of dentistry as an alternative to the widely used titanium has gained importance. Thereby, cellular response is dictated by the implant material and surface topography [8]. To examine the influence of material and surface topographies, osteoblasts and epithelial cells were cultivated on alumina (ATZ) and zirconia (TZP-A) ceramics in three modifications with rough titanium serving as a control. Since Mick et al. [22] found that acid etching does not improve the mechanical properties of glass ceramic coatings and Bächle et al. [11] asserted no additional effect on cellular behavior, this procedure was omitted in the present study. The native ceramic specimens with a smooth surface resulted in the lowest cell metabolic activity. This might have been caused by a lower cell attachment proven by large unsettled areas in the live/dead staining. Reduced cell adhesion on smooth surfaces was previously described [15]. In general, pointing to our material control, osteoblast adhesion and proliferation on rough titanium is ensured, pointing to the metabolic activity and live/dead staining, and has been proven in several works [1, 16, 17]. Our data exhibit the clearly increased metabolic activity of human osteoblasts on the roughened ceramic specimens coated with glass solder matrices. The advantages of ceramics, both alumina and zirconia, over titanium for osteoblasts' behavior* in vitro* were proven several times [5, 6, 8, 11, 25]. The synthesis of procollagen type 1 in human osteoblasts was increased on all ceramic specimens, mainly on modified ones, as mentioned by Depprich et al., so far [3]. Moreover, silicate-based bioglass seems to have stimulatory effects on osteoblast growth and differentiation [18]. Overall, rougher surfaces appear to be beneficial for enhanced osteoblast proliferation and adhesion [4, 8, 16, 26]. In our study, the gingival epithelial cells responded to the examined surfaces partially opposite to the osteoblasts and exhibited a slightly decreased viability and less dense cell layers on the native and S1 coated ceramic specimens, which is supported by several works [27, 28]. Nevertheless, cell viability was significantly increased on the S2 coated ceramic specimens. At the same time, the negative effects of titanium roughness on epithelial cells [21] and the preference for smooth titanium were described [28]. Continuative tests with human epithelial cells should be performed to verify the influence of materials and surface topographies. In further studies, the behavior of fibroblasts as stromal cells could also be determined. On the one hand, their adhesion and proliferation has been proven to be enhanced by rough surfaces [29, 30]. On the other hand, smoother surfaces are preferred [15], but not clearly specified for titanium or ceramic [5].

In subsequent studies, further data regarding cellular activity as alkaline phosphatase activity or expression of osteogenic marker proteins as osteocalcin should be gathered to confirm the present results. Furthermore, iterative tests with epithelial cells and also fibroblasts should be done determining collagen synthesis and GAG production. In addition, FESEM images of cell seeded ceramic specimens could be performed to analyse cell behavior or cell orientation on the different surfaces.

Apart from the cell biological results we found that ceramic specimens coated with glass solder matrices showed higher surface roughness values than those of the native references and sandblasted ceramics, which are comparable to sandblasted titanium, constituting a promising precondition for cellular response [31]. These findings are supported by field emission scanning electron microscopy images, which show the relatively plain surface of the native ceramic in its machined condition and in contrast the modified ceramic and titanium surfaces with comparable rough topographies.

The adhesive strength of both coating configurations is clearly below the tensile strength of the utilized bonding agent (approximately 100 MPa). However, the minimum adhesive strength of coatings as demanded by ASTM standard F-1147 is exceeded, which indicates a high stability of the glass solder matrix coatings. Furthermore, the necessary preconditioning of ceramic surfaces via sandblasting prior to coating with glass was proven not to have a negative influence on the bending strength of the samples. However, the coating process itself led to a significant decrease. Application of the pure heating process to TZP-A samples showed that this huge decrease is partly generated by the influence of temperature, which is consistent with the findings of Guazzato et al. [32]. The decline in bending strength may be indicated by the structural effects of glass solder particles diffusing into the ceramic base material. This should be investigated separately. Nevertheless, the remaining bending strength of about 700 MPa to 800 MPa is still within the range of commercially available zirconia [33].

## 5. Conclusion

The ceramic material with glass solder matrix coating was shown to be suitable for replacing titanium as a standard implant material in dental surgery regarding mechanical properties and enhanced osteoblastic metabolic activity and collagen synthesis. Studies with human epithelial cells have revealed similar results but should be performed with other human cells like fibroblasts.
